# Photochemically induced thrombosis combined with chronic restraint stress for modeling post-stroke depression in mice

**DOI:** 10.3389/fnins.2025.1547551

**Published:** 2025-02-28

**Authors:** Tumarisi Tuersunjiang, Qingchen Wang, Zhengzheng Wang, Feng Gao, Zhengchun Wang

**Affiliations:** ^1^The Affiliated People's Hospital of Ningbo University, Ningbo, Zhejiang, China; ^2^Zhejiang Key Laboratory of Pathophysiology, Basic Medical Sciences, Health Science Center, Ningbo University, Ningbo, Zhejiang, China

**Keywords:** photochemically induced thrombosis, chronic restraint stress, stroke, depression, mice model

## Abstract

**Introduction:**

Post-stroke depression (PSD) is a prevalent neuropsychiatric disorder associated with impaired recovery in stroke survivors, potentially linked to dysregulation of brain-derived neurotrophic factor (BDNF). This study aimed to establish a novel animal model of PSD by integrating ischemic brain injury with chronic psychological stress.

**Methods:**

Mice were subjected to photochemically induced thrombosis (PIT) to generate focal ischemic lesions in the parietal lobe, followed by chronic restraint stress (CRS) to simulate post-stroke psychological stress. Behavioral assessments (sucrose preference test, forced swim test, tail suspension test) and molecular analyses (BDNF, synaptophysin [SYP], interleukin-1 [IL-1], tumor necrosis factor-*α* [TNF-*α*]) were conducted to evaluate depressive-like phenotypes and neuroinflammatory markers.

**Results:**

The PIT model produced consistent ischemic damage, with an average infarct area of 2.580 ± 0.426% in the parietal lobe. Mice exposed to PIT-CRS exhibited significant depressive-like behaviors, including reduced sucrose preference (*p* < 0.001), increased immobility time in the forced swim test (*p* = 0.056), and prolonged immobility in the tail suspension test (*p* = 0.168) compared to the Sham group. Molecular analyses revealed marked downregulation of BDNF (*p* = 0.004) and SYP (*p* = 0.074), alongside upregulated IL-1 (*p* = 0.024) and TNF-α (*p* = 0.368) levels in the PIT-CRS group.

**Conclusion:**

The PIT-CRS model provides a comprehensive and reproducible platform for studying PSD. By integrating both ischemic injury and chronic stress, this model captures the multifaceted nature of PSD and offers valuable insights into its pathophysiology. Future research using this model could pave the way for the development of targeted therapies for PSD.

## 1 Introduction

Post-stroke depression (PSD) is a severe psychiatric condition that emerges in a substantial number of stroke survivors (Kaneko et al., 1985; Umemura et al., 1993). It typically occurs within weeks or months after a stroke, manifesting symptoms such as psychomotor agitation, feelings of worthlessness, sleep disturbances, weight changes, and suicidal thoughts (Fujie et al., 1990). Research indicates that the prevalence rates of PSD widely vary, especially in regions like China, where it can range from 10 to 68% (Deng et al., 2024). PSD not only diminishes a patient’s quality of life and recovery but also contributes to higher mortality rates and imposes considerable strain on families and healthcare systems.

In studying PSD, animal models play a crucial role in investigating the pathophysiological mechanisms and testing potential treatments. The middle cerebral artery occlusion (MCAO) model is frequently employed for this purpose (Kaneko et al., 1985; Umemura et al., 1993; Fujie et al., 1990), though it often presents challenges, such as low survival rate and significant neurological deficits, which may render them unsuitable for accurately assessment of depression-like behaviors. Additionally, existing models often demonstrate considerable variability in the size and location of damage influenced by different induction methods and individual animal responses (Majid et al., 2000; Nagai et al., 2010; Zhang et al., 2010). This variability in the initial extent of damage can impact the intensity of repair responses, and with larger injuries typically triggering more robust reparative mechanisms (Nagai et al., 2010).

To address these issues, a PIT-CRS model has been developed that induces consistent ischemic damage in the cortex without resulting in behavioral disturbances. This model utilizes photochemically induced thrombosis (PIT) in the parietal lobe (Watson et al., 1985) combined with chronic restraint stress (CRS) (Li et al., 2013), simulating the physical constraints that many stroke patients experience. The current model ensures high reproducibility in the area and extent of damage (Zhang et al., 2012), thereby reducing the risk of confounding behavioral effects.

## 2 Materials and methods

### 2.1 Animal maintenance and housing

Eight-week-old male BALB/c mice (20–25 g, ZheJiang VitalRiver Laboratory Animal Technology) were kept under a 12 h light/dark cycle with ad libitum access to food and water. All experimental procedures were approved by the Committee on Animal Care of Ningbo University (Permit number: 13763) and conducted in accordance with institutional, national guidelines, and regulations. Male BALB/c mice were selected for their low hormonal variability and established reliability in stroke models (Majid et al., 2000; Tsuchimine et al., 2020; Sathyanesan et al., 2017).

### 2.2 Focal cerebral infarction induced by PIT

Focal cerebral infarction induced by photochemically induced thrombosis (PIT) results in localized necrosis of brain tissue. This occurs as a specific photochemical reaction initiates thrombus formation, obstructing cerebral blood flow. This model provides a controlled setting for investigating the pathological mechanisms of stroke and its effects on brain function (Nagai et al., 2010; Watson et al., 1985; Yano et al., 2011; Lee et al., 2007; Shakova et al., 2021; Pawletko et al., 2022).

Ischemic brain injury was induced using a modified photochemically induced thrombosis (PIT) method (Nagai et al., 2010; Yano et al., 2011). Briefly, mice were anesthetized using 3.5% isoflurane and then firmly secured in a stereotaxic apparatus. The fur on the head was carefully removed, and a midline incision was made to expose the skull. The left parietal cortex located at the coordinates AP: +1.2 mm and ML: +1.0 mm, was precisely identified and marked. A circular target area with a diameter of 2.5 mm was drilled until clear blood vessels were visible. A sterilized black plastic sheet with a matching circular hole was placed over the skull, ensuring that the hole was positioned directly above the target cortical region while covering the other areas. An optic fiber with a diameter of 1.0 mm was then placed directly on the thinned skull.

The mice were maintained on a heated pad at a temperature of 37 ± 0.5°C. A solution of Rose Bengal sodium salt (G8540, Solarbio, Beijing, China) at a concentration of 20 mg/mL was injected into the tail vein at a volume of 0.1 mL/kg. Immediately after injection, the target area was illuminated with green light (532 nm, 40 mW/cm^2^) for 10 min using an optic fiber connected to a light source (model EG71525; MGL-III-532-100 mw, Changchun Leirui Optoelectronics Technology company, China). Following laser exposure, the skin was sutured, and the mice were closely monitored on the heating pad until they fully regained consciousness before being returned to their home cages. Neurological function was evaluated 1 day after PIT using a Modified Neurological Severity Score assessment (mNSS) (Chen et al., 2001).

### 2.3 Depression induced by CRS

Chronic restraint stress (CRS) involves subjecting animals to a physically restrictive environment for an extended period, severely limiting their movement (McEwen, 2007; Lee et al., 2022). This sustained stress can trigger a range of psychological and physiological alterations, including the development of anxiety, depression, and changes in neuroendocrine function. When combined with photochemically induced thrombosis (PIT), this approach enables a comprehensive investigation of the mechanisms underlying post-stroke depression and provides insights into potential therapeutic strategies.

In this study, mice were subjected to CRS as described (Chiba et al., 2012), with minor modifications. Briefly, mice were individually restrained in ventilated hand-made polypropylene tubes (inner diameter 3 cm × length 10 cm; RWD Life Science, Cat# 68031) for 14 consecutive days. Restraint duration was progressively increased as follows: 5 h (days 1–2), 6 h (days 3–5), 7 h (days 6–9), and 8 h (days 10–14). All restraints were initiated at 09:30 h to minimize circadian variability. To ensure adequate ventilation and normal respiration, multiple small holes (approximately 0.5 cm in diameter) were uniformly drilled in both the body and the lid of the restraint tube.

### 2.4 Behavioral assessment

A series of behavioral assessments, including the open field test, sucrose preference test, forced swim test, and tail suspension test, were conducted to evaluate depressive-like symptoms in the mice.

#### 2.4.1 Open field test

The open field test (OFT) evaluates locomotor activity and anxiety in mice (Seibenhener and Wooten, 2015; Prut and Belzung, 2003). Mice were individually placed in a 40 cm × 40 cm × 40 cm acrylic arena for a 10 - min period. After each trial, the arena was thoroughly disinfected with 75% ethanol to prevent olfactory cues from influencing subsequent tests. Movements were recorded using an infrared camera, and the data were analyzed using the ANY-maze system. The arena was cleaned with 75% ethanol between trials to eliminate odor cues.

#### 2.4.2 Sucrose preference test

The sucrose preference test (SFT) was employed to evaluate changes in hedonic responses (Willner et al., 1987). The test consisted of a 72-h acclimation phase followed by a 12-h testing phase. During acclimation, mice were provided with two bottles of 1% sucrose solution. Subsequently, one bottle was replaced with plain water. To prevent position-based memory effects, the positions of the bottles were switched every 12 h during the acclimation phase and every 3 h during the testing phase. The sucrose preference index was calculated as follows: [sucrose consumption/(sucrose consumption + plain water consumption)] × 100%.

#### 2.4.3 Tail suspension test

The tail suspension test (TST) measured the severity of depression by recording the duration of immobility (Steru et al., 1985). Mice were first acclimated for 30 min. Then, they were suspended 45 cm above the ground for 6 min, and the duration of immobility during the last 4 min was observed and recorded.

#### 2.4.4 Forced swim test

The forced swim test (FST) evaluated the level of despair in the mice (Porsolt et al., 1977). After a 30-min acclimation period, mice were placed in water at 24°C for a 6-min swim. Immobility, defined as floating or minimal movement, was measured. After the test, the mice were dried and returned to their cages.

### 2.5 Brain infarct measurement

To detect infarcts in the mouse brain after PIT, mice were anesthetized with isopentobarbital sodium (100 mg/kg, Shanghai Pharma New Asia Pharma, China). The brains were removed at 1 and 14 days post-stroke. The removed brains were rapidly frozen at −20°C for 20 min and then sliced into five 2-mm coronal sections using a mold and a stainless-steel blade. TTC staining and Nissl staining were used to assess the infarct size (Chu et al., 2018).

For TTC staining, the slices were incubated in 2% 2,3,5-triphenyltetrazolium chloride (TTC) at 37°C for 30 min in the dark. Subsequently, they were immersed in 4% paraformaldehyde for 12 h, and photographs were taken to document the results. For Nissl staining (Nagai et al., 2010; Yano et al., 2011), mice were deeply anesthetized with isopentobarbital sodium and perfused with saline, followed by fixation in 4% paraformaldehyde. The brain tissues were dehydrated and embedded in paraffin. Using a microtome, the tissues were sliced into 5-μm sections and placed on glass slides. The sections were deparaffinized in a laboratory oven and rehydrated through a series of decreasing ethanol concentrations. They were then immersed in Nissl stain solution (G1430, Solarbio, China) for 30 min. After a second dehydration step using increasing ethanol concentrations, the sections were mounted with neutral resin for microscopic examination.

### 2.6 Enzyme-linked immunosorbent assay

The concentrations of IL-1 and TNF-*α* were quantified using commercial ELISA kits (Jiangsu Kete Biological Technology Co., Ltd.). Tissue samples were weighed, homogenized in phosphate-buffered saline (PBS), and centrifuged at 1,000 × g for 10 min at 4°C. Supernatants were collected and stored at −80°C until analysis. Three samples per group were analyzed using the IL-1 (KT2040-A) and TNF-α (KT2132-A) kits.

### 2.7 Western blot

Mice were anesthetized with pentobarbital (50 mg/kg). Hippocampus tissue was lysed in SDS buffer, heated at 95°C for 5 min. Protein in samples (45 μg) from Sham, and PIT-CRS groups (quantified by BCA) was loaded on 10 and 12% SDS-polyacrylamide gels. After electrophoresis, proteins were transferred to a BioRad nitrocellulose membrane and blocked with 5% non-fat milk. Primary antibodies for BDNF (1:1,000, db15513, Dakewe, China) and SYP (1:20,000, 17,785-1-AP, Proteintech, China) were applied overnight at 4°C, followed by an Alexa Fluor 800-conjugated secondary antibody for 120 min. Target bands were detected and quantified using a Tanon-5300 M system and ImageJ. Each protein was analyzed in triplicate.

### 2.8 Statistical analysis

GraphPad Prism 8.0 (GraphPad Software, San Diego, CA) was used for statistical analyses. Unpaired Student’s *t*-test was used for two-group comparisons, and one-way ANOVA with Tukey’s test was used for more than two groups. All tests were two-sided; *p* < 0.05 was considered significant.

## 3 Results

### 3.1 Brain damage assessment in the PIT model using TTC and Nissl staining

**Figure 1:**
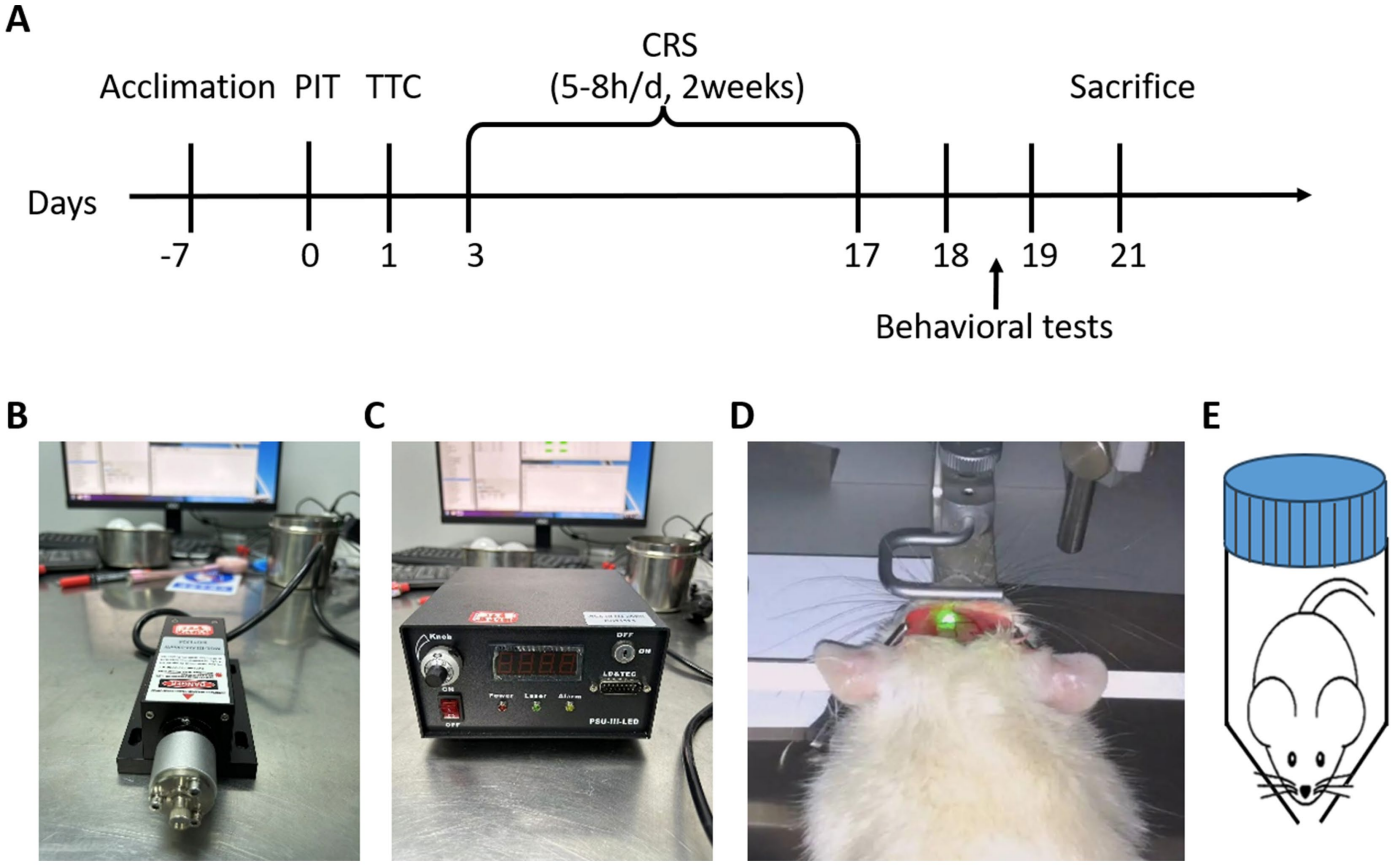
Experimental design and procedure. **(A)** Experimental timeline. After one-week acclimation, photochemically-induced thrombosis (PIT) surgery was performed to induce focal cerebral ischemia, simulating a stroke event. On day 1 post-surgery, triphenyltetrazolium chloride (TTC) staining was conducted to assess tissue ischemia-induced damage. Chronic restraint stress (CRS) was then applied for 2 weeks (5–8 h/day) to simulate a chronic stress environment, known to induce depressive-like behaviors. Behavioral tests were performed to evaluate depression-related behaviors, and animals were sacrificed following behavioral tests. **(B,C)** Laser source and control unit: 532 nm, 40 mW/cm^2^, 10 min. **(D)** The mouse’s head was fixed to ensure precise exposure of the targeted brain area to the fiber-optic cable, enabling accurate induction of thrombosis in cerebral blood vessels. **(E)** CRS setup. A hand-made cylindrical restraint device was used to restrict movement while allowing free breathing.

**Figure 2:**
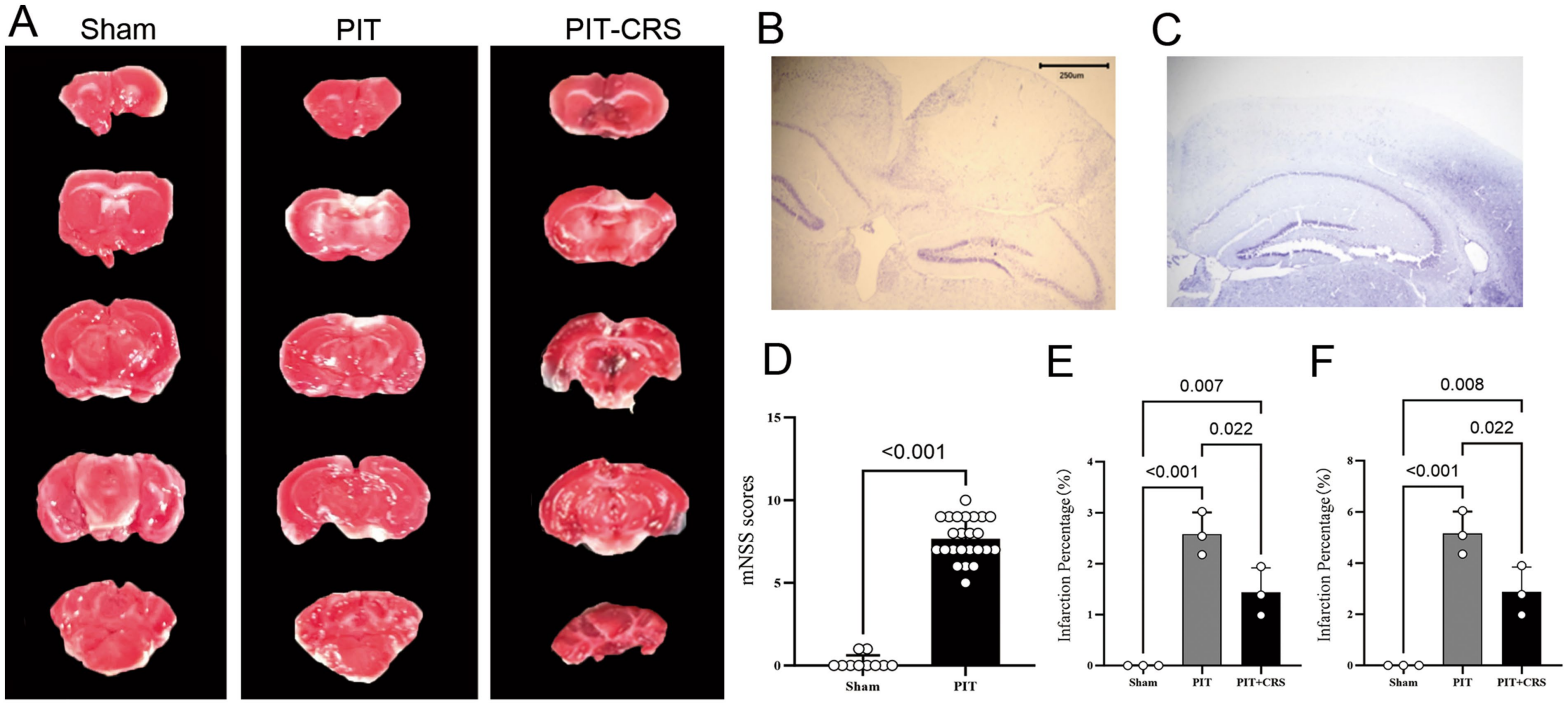
Effects of partial ischemic treatment (PIT) on mouse cortical tissue. **(A)** Representative images of 2,3,5-triphenyltetrazolium chloride (TTC)-stained coronal brain slices after 1 day of photochemically induced thrombosis (PIT), and 14 days of CRS after PIT (PIT-CRS), Scale bar: 10 mm. **(B,C)** Representative images of Nissl staining after PIT. **(D)** Modified Neurological Severity Score assessment (mNSS). **(E)** Percentage of infarct area. **(F)** Percentage of infarct volume.

This model utilizes photochemically induced thrombosis (PIT) in the parietal lobe, combined with chronic restraint stress (CRS). The overview of the experimental design and procedure was shown in the Figure 1. The results of TTC staining indicated a significant infarct area in the left cortex of PIT mice (Figure 2A), as evidenced by the pale regions in the stained sections. Nissl staining revealed marked neuronal damage in the infarcted regions, suggesting significant neurodegeneration. In contrast, the contralateral hemispheric cortex showed minimal infarction (Figures 2B,C). The modified Neurological Severity Score assessment (mNSS) of the PIT group was significantly higher than the Sham group (*t* = 17.985, *p* < 0.001) (Figure 2D). Particularly, the percentage of infarct area and infarct volume 1 day after PIT was significantly higher than the Sham group [*F*(2, 6) = 36.587, *p* < 0.001 for infarct area; *F*(2, 6) = 36.587, *p* < 0.001 for infarct volume]. The percentage of infarct area and infarct volume in PIT-CRS group was significantly higher than the Sham group [*F*(2, 6) = 35.59, *p* = 0.007 for infarct area; *F*(2, 6) = 36.587, *p* = 0.008 for infarct volume], while significantly lower than the PIT group [*F*(2, 6) = 36.587, *p* = 0.022 for infarct area; *F*(2, 6) = 36.587, *p* = 0.022 for infarct volume] (Figures 2E,F). These findings highlight the detrimental effects of the induced ischemia post PIT.

### 3.2 Behavioral tests reveal depression-like phenotype in the PIT-CRS model

**Figure 3:**
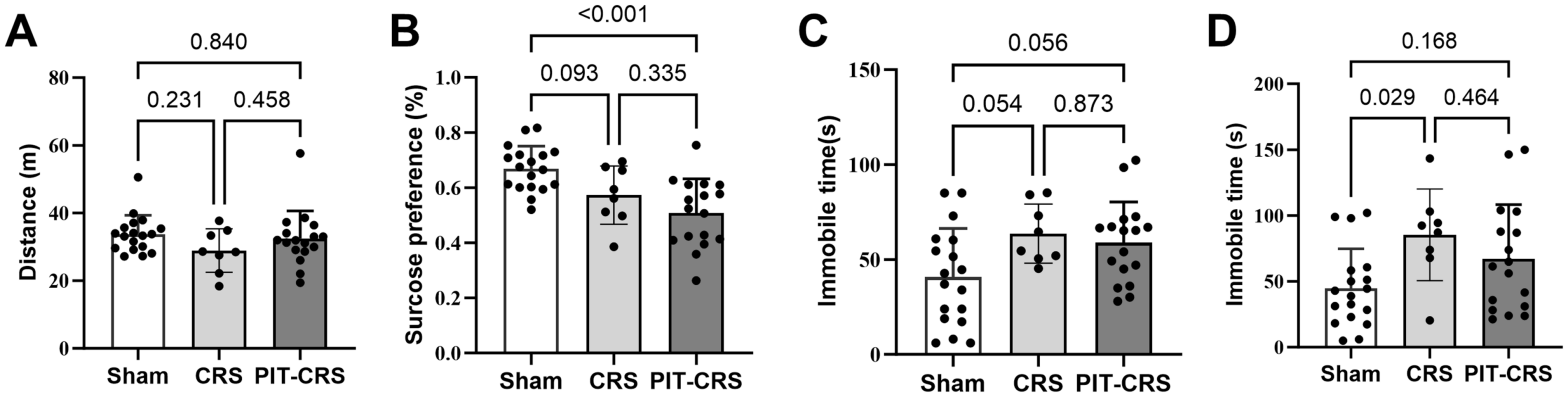
Behavioral tests confirm depression-like phenotype. **(A)** Movement distance is similar among the Sham, CRS and PIT-CRS groups in the open field test. **(B)** The percentage of sugar consumption for Sham, CRS and PIT-CRS groups in the sucrose preference test. **(C)** Immobility time in the forced swim test for Sham, CRS and PIT-CRS groups. **(D)** Immobility time in the tail suspension test (TST) for Sham, CRS and PIT-CRS groups. Data are presented as mean ± SEM, *n* = 18 for Sham group, *n* = 8 for CRS group, *n* = 17 for PIT-CRS group.

The results from behavioral tests demonstrated a pronounced depressive phenotype in the PIT-CRS model (Frank et al., 2022). There was no significant difference among the Sham, CRS and PIT-CRS groups in the locomotion activity in the OFT [*F*(2, 40) = 1.389, *p* = 0.261] (Figure 3A). The sucrose preference index of the CRS group exhibited a tendency lower than that of the Sham group [*F*(2, 40) = 10.271, *p* = 0.093], while the sucrose preference index of the PIT-CRS group was significantly higher than that of the Sham group [*F*(2, 40) = 10.271, *p* < 0.001] (Figure 3B). In the FST, the CRS group had longer immobile time than the Sham group [*F*(2, 40) = 4.123, *p* = 0.054], while the PIT-CRS group showed a tendency to influence the immobile time [*F*(2, 40) = 4.123, *p* = 0.056] (Figure 3C). In the TST, the CRS group had longer immobile time than the Sham group [*F*(2, 40) = 3.960, *p* = 0.029], while the PIT-CRS group showed no significant effects on the immobile time [*F*(2, 40) = 3.960, *p* = 0.168] (Figure 3D).

### 3.3 Dysregulated inflammatory and synaptic markers in the PIT-CRS model

The pathogenesis of PSD is complex, involving various factors such as lesion location, biogenic amines, cytokine inflammation, and gene polymorphisms (Frank et al., 2022). BDNF is considered a clinical biomarker for predicting PSD onset, while neuroinflammation and synaptic damage are key pathological changes linked to ischemic stroke (Shan et al., 2021; Zhang et al., 2024; Ramos-Cejudo et al., 2015; Zhang et al., 2024). We investigated BDNF levels, synaptic proteins, and inflammatory response factors in both groups.

**Figure 4:**
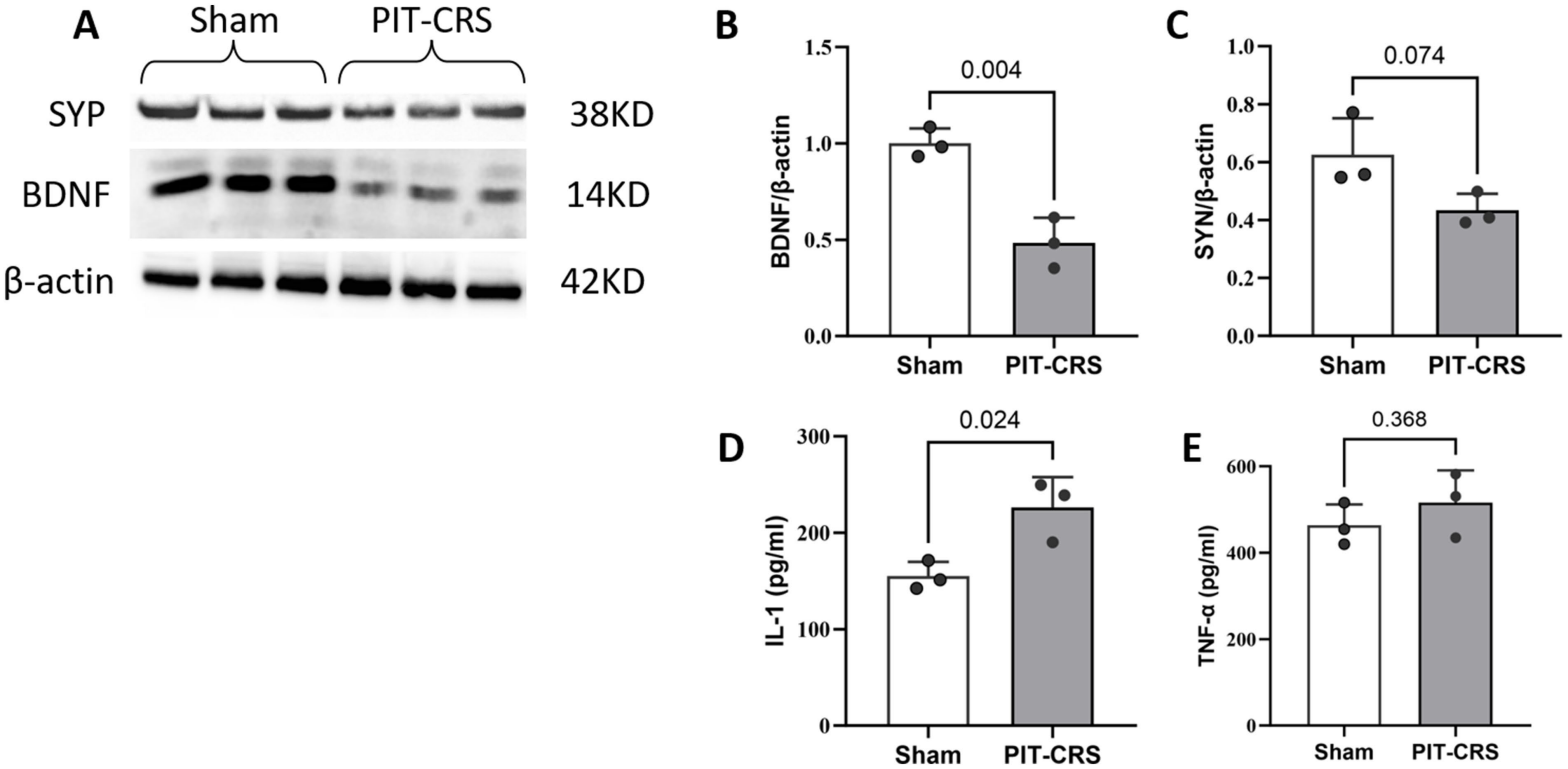
Synapse impairments and neuroinflammation in PIT-CRS model. **(A)** Western blotting of BDNF and SYP levels in the hippocampus of Sham and PIT-CRS mice. Quantification of **(B)** BDNF levels, **(C)** SYP levels, **(D)** IL-1 levels, and **(E)** TNF-*α* levels in the hippocampus of Sham and PIT-CRS mice. Each sample corresponds to an individual animal, with data derived from three different mice in each group. Data are presented as mean ± SEM, unpaired *t*-test.

The results indicate a significant decrease in BDNF levels in the PIT-CRS group compared to the Sham group (*p* < 0.01, *t* = 5.878, *df* = 4). Additionally, in the PIT-CRS model, SYP expression trended downwards compared to the Sham group, but not significantly (*p* = 0.074, *t* = 2.404, *df* = 4). Furthermore, there was an increase in IL-1 expression IL-1 (*p* = 0.024, *t* = 3.532, *df* = 4) and a minor trending increase in TNF-*α* (*p* = 0.368, *t = 1.014, df = 4*) compared to the Sham group (Figure 4).

## 4 Discussion

Animal models are critical for understanding the pathophysiology of post-stroke depression (PSD) and evaluating potential therapeutic interventions. In this study, we developed a PIT-CRS model by combining photochemically induced thrombosis (PIT) with chronic restraint stress (CRS). This combined approach effectively simulates both the neurological damage and psychological stress associated with PSD, thus laying a solid groundwork for exploring its underlying mechanisms and potential treatments.

The PIT component of the model allows precise induction of focal ischemia in specific brain regions, such as the parietal lobe, enabling targeted study of their roles in PSD (Fukumoto et al., 2012; Morishita et al., 2020; Matsuno et al., 1993; Yao et al., 2024). This method is highly reproducible and closely mimics the ischemic damage observed in human stroke, offering valuable insights into stroke-related pathology. However, the surgical nature of PIT introduces potential stressors, such as anesthesia and postoperative pain, which must be carefully managed to minimize confounding effects. On the other hand, CRS provides a simple yet effective means of simulating chronic psychological stress, a key contributor to PSD. By combining PIT and CRS, our model captures both the neurological and psychological dimensions of PSD, enhancing its relevance and applicability.

A key strength of this model is its ability to replicate the neurobiological changes associated with PSD. We observed a significant reduction in BDNF levels in the PSD group compared to the Sham group, consistent with the role of BDNF as a critical biomarker for PSD (Shan et al., 2021). Additionally, the PIT-CRS model exhibited elevated levels of inflammatory factors in the hippocampus, supporting the growing evidence linking neuroinflammation to depressive disorders (Zhang et al., 2024; Iob et al., 2020; Munshi et al., 2024). Furthermore, synaptic integrity was compromised, as indicated by a significant decline in synaptic protein SYP levels, aligning with previous findings on synaptic dysfunction in depression (Jiang et al., 2023). These results underscore the utility of our model for studying the molecular and cellular mechanisms underlying PSD.

When comparing PIT-CRS and MCAO-CRS models for PSD, key differences in replicability and physiological relevance appear. PIT precisely induces focal ischemia by controlled photochemical reactions, mimicking localized strokes consistently. MCAO, affected by surgical skill and anatomy, causes inconsistent infarcts and less-targeted damage. Both models target chronic stress in PSD. But high MCAO surgery mortality in mice disrupts CRS implementation. The less-invasive PIT in our model reduces mortality, ensuring more reliable stress exposure. The PIT-CRS model combines local ischemia and chronic stress to mimic human PSD. MCAO models, useful for stroke research, often lack controlled local ischemia and sufficient chronic stress for full PSD replication.

Despite the advantages of our combined PIT and CRS model, it has certain limitations. One of the main challenges is the inability to fully account for individual differences among mice. Variability in individual responses to ischemia and restraint stress may affect the consistency of results. Differences in surgical outcomes and stress tolerance could lead to variations in the expression of depression-like behaviors, highlighting the need for careful standardization of experimental conditions. Future studies should explore strategies to minimize these variations, such as optimizing surgical protocols and stress regimens, to improve the reliability of the model.

In conclusion, the PIT-CRS model provides a comprehensive and reproducible platform for studying PSD. By integrating both ischemic injury and chronic stress, this model captures the multifaceted nature of PSD and offers valuable insights into its pathophysiology. Future research using this model could pave the way for the development of targeted therapies for PSD.

## Data Availability

The original contributions presented in the study are included in the article/Supplementary material, further inquiries can be directed to the corresponding authors.
