# Supplementary figures and images for: Photochemically induced thrombosis combined with chronic restraint stress for modeling post-stroke depression in mice
Source: Front Neurosci. 2025 Feb 28;19:1547551. doi: 10.3389/fnins.2025.1547551 (PMC11906474; doi:10.3389/fnins.2025.1547551)

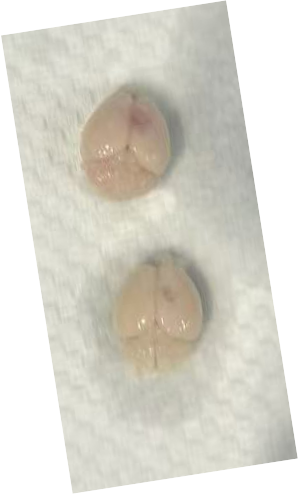

Supplement: Supplementary file 1 [file Image_1.tif]
